# Interconnections between the Oral and Gut Microbiomes: Reversal of Microbial Dysbiosis and the Balance between Systemic Health and Disease

**DOI:** 10.3390/microorganisms9030496

**Published:** 2021-02-26

**Authors:** Brandon Khor, Michael Snow, Elisa Herrman, Nicholas Ray, Kunal Mansukhani, Karan A. Patel, Nasser Said-Al-Naief, Tom Maier, Curtis A. Machida

**Affiliations:** 1 Academic DMD Program, Oregon Health & Science University, 2730 SW Moody Avenue, Portland, OR 97201, USA; khor@ohsu.edu (B.K.); snowmi@ohsu.edu (M.S.); herrman@ohsu.edu (E.H.); rayni@ohsu.edu (N.R.); mansukha@ohsu.edu (K.M.); patekara@ohsu.edu (K.A.P.); 2 Department of Integrative Biomedical and Diagnostic Sciences, School of Dentistry, Oregon Health & Science University 2730 SW Moody Avenue, Portland, OR 97201, USA; saidalna@ohsu.edu (N.S.-A.-N.); maiert@ohsu.edu (T.M.)

**Keywords:** oral microbiome, gut microbiome, microbial dysbiosis, inflammatory disorders, therapeutics, precision medicine, systemic health and disease

## Abstract

The human microbiota represents a complex array of microbial species that influence the balance between the health and pathology of their surrounding environment. These microorganisms impart important biological benefits to their host, such as immune regulation and resistance to pathogen colonization. Dysbiosis of microbial communities in the gut and mouth precede many oral and systemic diseases such as cancer, autoimmune-related conditions, and inflammatory states, and can involve the breakdown of innate barriers, immune dysregulation, pro-inflammatory signaling, and molecular mimicry. Emerging evidence suggests that periodontitis-associated pathogens can translocate to distant sites to elicit severe local and systemic pathologies, which necessitates research into future therapies. Fecal microbiota transplantation, probiotics, prebiotics, and synbiotics represent current modes of treatment to reverse microbial dysbiosis through the introduction of health-related bacterial species and substrates. Furthermore, the emerging field of precision medicine has been shown to be an effective method in modulating host immune response through targeting molecular biomarkers and inflammatory mediators. Although connections between the human microbiome, immune system, and systemic disease are becoming more apparent, the complex interplay and future innovations in treatment modalities will become elucidated through continued research and cross-disciplinary collaboration.

## 1. Introduction

The human microbiota contains an extensive variety of microbial organisms ranging from bacteria and viruses to archaea, protozoa, and fungi. This diverse and complex community of microbes help to dictate the balance between homeostatic health and pathology, providing important functional and immunologic benefits to the host in eubiosis or contributing to the etiopathogenesis of dysbiotic diseases [1]. Gastrointestinal (GI) and oral microbial dysbiosis have been associated with several devastating illnesses including caries, periodontitis, systemic lupus erythematosus (SLE), rheumatoid arthritis (RA), and colorectal cancer (CRC). In addition, evidence suggests that translocation of oral microbes to the gut can play a significant role in the development of GI disease, emphasizing the importance of this topic for future therapeutic innovation and prophylactic action. Current treatments aimed at rectifying dysbiosis, such as fecal microbiota transplantation (FMT), probiotics, prebiotics, and synbiotics, have been shown to be effective, but represent more indiscriminate approaches to therapy. Technological advances have allowed for a more intimate understanding of disease processes at an individual level, elucidating targets that can be pursued for a more personalized approach to treatment. Precision medicine focuses on exploiting these individualized factors to treat disease. Subsequently, in this review, we describe the interconnections between the oral and GI microbiota, their impact on health and disease, treatments to correct dysbiosis, and targeted therapies to reverse dysbiosis in the context of the emerging field of precision medicine.

## 2. Composition of Oral Microbiome and Its Impact on Health and Disease

The existence of complex oral maxillofacial anatomical structures in the head and neck provides several ecological niches for bacterial colonization [2]. Factors such as nutrient availability, host immune exposure, oxygen content, and temperature dictate bacterial localization [3]. As the host matures, the oral microbiota evolves through several stages, usually acquiring *Streptococcus* as pioneer colonizers before population by other oral inhabitants [4]. Though large inter-individual variation often exists, a “core taxa” of oral microbes comprising the phlya Bacteroidetes, Firmicutes, Actinobacteria, Proteobacteria, and Fusobacteria is commonly shared among different individuals [5]. A snapshot of health reveals the presence of *Streptococcus mitis* on the buccal mucosa, *Streptococcus salivarius* in the saliva and on the dorsal tongue, and *Streptococcus sanguinis* colonizing tooth surfaces. These bacteria alter their environment by modulating pH, nutrient availability, and other factors which, in turn, sets the stage for subsequent microbial colonization. As time progresses, more complex bacterial communities develop, and a homeostatic balance of microorganisms becomes established within their respective niches. Although the *Streptococcus* genus typically dominates the majority of oral surfaces, other bacterial species such as *Fusobacterium* spp. inhabit areas more conducive to their survival, including the subgingival biofilm. Upon microbial homeostasis of the mouth, the phylotypes of *Streptococcus*, *Granulicatella*, *Neisseria*, *Haemophilus*, *Corynebacterium*, *Rothia*, *Actinomyces*, *Prevotella*, *Capnocytophaga*, *Porphyromonas*, and *Fusobacterium* all predominate in health [6]. However, when oral conditions shift, this microbial balance is disrupted, elevating the levels of specific bacterial species, which in turn encourages the pathogenesis of various diseases including dental caries, periodontitis, and endodontic infections.

## 3. Microbial Dysbiosis and Development of Oral and Systemic Diseases

According to the United States Centers for Disease Control and Prevention, dental caries represents the most prevalent chronic ailment of adolescents and children aged 6–11. Dental caries is characterized by degradation from bacterial acids, which results in decay and loss of tooth structure. With the introduction of refined flour and sugar, *S. mutans* developed the ability to resist higher levels of oxidative stress and a capacity to thrive despite elevated levels of carbohydrate-derived acid metabolites, effectively allowing them to outcompete other less cariogenic bacteria and change the homeostatic balance of the oral microbiota [7]. With its newfound niche and abilities, *S. mutans* became a prominent force in driving the pathogenesis of caries and was once considered to be its primary etiology. However, newer studies have implicated other bacteria in the pathogenesis of carious lesions, where 10–20% of individuals with caries demonstrate non-detectable levels of *S. mutans* [8]. Carious lesions initiated by *S. mutans* tend to contain microbes from the *Lactobacillus*, *Propionibacterium*, and *Atopobium* genera. The absence of *S. mutans*, however, is replaced with the presence of significantly-elevated levels of low pH-tolerant, non-*S. mutans* streptococci, *Bifidobacterium dentium*, and *Lactobacillus* species. *Scardovia wiggsiae* has been strongly associated with severe early childhood caries (S-ECC), an advanced form of the disease that affects primary dentition, in the presence or absence of *S. mutans*, and is found in over 50% of children that have S-ECC [9].

Periodontitis, while sharing a similar dysbiotic organization as caries, follows different pathways and mechanisms of etiopathogenesis and progression [10]. A landmark study in 1998 by Socranksy and co-workers reported that periodontitis was best represented through complexes of bacteria rather than a single etiologic agent [11]. While bacteria such as *Aggregatibacter actinomycetemcomitans*, *Tannerella forsythia*, and certain *Prevotella* and *Treponema* species have been implicated in periodontitis [4,11], specific bacterial combinations are recognized as better indicators of disease, with the most well-known being the “red complex” consisting of *Porphyromonas gingivalis*, *T. forsythia*, and *Treponema denticola* [11].

Upon primary infection of the dental pulp, commonly observed microorganisms consist of *Peptostreptococcus, Dialister, Parvimonas micra, Fusobacterium nucleatum, Filifactor alocis, T. denticola, Pseudoramibacter alactolyticus, Porphyromonas endodontalis, P. gingivalis, Prevotella nigrescens, Prevotella baroniae, Prevotella intermedia, *and* T. forsythia,* with significant but lower levels of *Enterococcus faecalis* [12]. However, with root canal treatment and retreatment, elevated levels of *E. faecalis, F. alocis, P. alactolyticus, P. micra, Propionibacterium propionicus, Streptococcus constellatus, *and* Streptococcus anginosus* have been detected [12]. Table 1 summarizes the findings of several reports describing the importance of several oral microorganisms according to oral health status.

Oral bacteria are also classically implicated in the pathogenesis of atherosclerotic plaques including: *T. forsythia*, *P. gingivalis*, *A. actinomycetemcomitans*, and *P. intermedia*. *P. gingivalis* stimulates epithelial production of IL-6, INF-γ, and TNF-α which leads to local inflammatory processes that degrade oral gingival tissue and subsequently allow for bacterial access into the vasculature [13]. This auto-destruction of the oral tissue–blood barrier allows for dissemination of bacteria and their byproducts into the bloodstream, and enables access to coronary atherosclerotic plaques. Bacteria found in atherosclerotic plaques also form complex biofilms that mirror dental plaques and consist of three stages of colonization with *F. nucleatum* serving as a bridging species [14].

Some oral bacteria implicated as causative agents of pneumonia include *P. gingivalis*, *P. intermedia*, *A. actinomycetemcomitans*, *Capnocytophaga*, *Eikenella corrodens*, and *S. constellatus* [15]. Oral pathogens are thought to play two indirect roles in the pathogenesis of pneumonia: modification of the oral cavity’s innate immunity and cytokine production. Enzyme secretion caused by periodontal pathogens degrades mucins and the salivary pellicle. This reduces the body’s ability to clear pathogenic respiratory bacteria from the mouth and also exposes adhesion sites that allow them to bind to structures in the oral cavity [16]. The cytokines produced by the oral immune response to periodontal bacteria (e.g., IL-1α, IL-1β, IL-6, IL-8, TNF-α) can be aspirated and travel to the lower respiratory tract. Once in the lower respiratory tract, these cytokines can cause recruitment of inflammatory cells that damage respiratory epithelium and increase susceptibility to respiratory pathogen colonization [16].

## 4. Gut Microbiome and Its Impact on Systemic Health and Disease

While the oral microbiota presents a rich and diverse source of microbial species, it is second in abundance and diversity to the gut microbiota, exceeding 10^14^ microorganisms [17]. Similar to the oral cavity, gut bacterial colonization occurs shortly after birth, taking up surrounding microbes from the mother’s vagina, feces, skin, and saliva [18]. As the host matures, environmental interactions and host physiology help to establish this complex ecosystem, which stabilizes over time [18]. Collectively, this set of microorganisms live symbiotically within their host, reaching a healthy homeostatic balance known as eubiosis. In eubiosis, these microbial communities perform necessary functions such as nutrient conversion, vitamin formation, and immune tolerance [19]. In addition, a eubiotic gut microbiota aids in the maintenance of hepatic health [20] and can ward off neurological diseases that stem from communication between the enteric and central nervous systems [21], highlighting the importance of GI eubiosis to overall health.

A healthy gut microbiota consists mainly of the phyla Firmicutes (30–50%), Bacteroidetes (20–40%), Actinobacteria (1–10%), and, to a lesser extent, Proteobacteria [18]. Three robust bacterial GI clusters, also known as enterotypes, are understood to exist [19]. Enterotype 1, associated with the heavily carbohydrate, fat, and protein-based diets of Western cultures, is enriched with *Bacteroides* and *Parabacteroides* [22]. Enterotype 2, composed mainly of *Prevotella* and *Desulfovibrio*, concentrates around those with high-fiber diets rich in vegetables and fruits [22]. Enterotype 3, mainly dominated by *Ruminococcus* and *Akkermansia*, is the most frequent enterotype. An important functional aspect of GI health is the production of short-chain fatty acids (SCFAs) such as butyrate, acetate, and propionate mainly from the phyla Firmicutes and Bacteroidetes [23]. Butyrate serves as the primary energy source for colonic epithelial cell maintenance and is involved in the expansion and differentiation of regulatory T cells that modulate immune activity [23]. Some bacteria also exhibit indirect mechanisms that limit pathogen presence. *Bacteroides thuringiensis* secretes a bacteriocin that targets certain Bacilli and Clostridia, such as *Clostridioides difficile*. Others express lipopolysaccharide and flagellin, stimulating the immune system through toll-like receptors (TLRs) and priming the immune response to ward off unwelcome guests [23]. Healthy bacteria also directly compete for nutritional and physical niches, allowing for “colonization resistance” against pathogens [23].

## 5. Microbial Dysbiosis in the Gastrointestinal System and Metabolic Triggers of Systemic Diseases

An outgrowth of Proteobacteria and a generalized decrease in bacterial diversity characterize microbial dysbiosis in the gut [19]. Since Bacteroidetes and Firmicutes represent a large proportion of the GI microbiota, these populations require more significant shifts to cause pathology, whereas modest increases in marginalized bacteria may exhibit more profound effects [19]. Moderate shifts of microorganisms within the GI system resulting from nutritional changes, antibiotics, chemotherapy, or other environmental factors allow aggravating elements such as oxidative stress to exacerbate alterations in specific bacterial groups, one example being Enterobacteriaceae, whose numbers are altered by oxidative inflammation [19]. Another consequence of dysbiosis is increased intestinal permeability, which is associated with the high-fat diets seen in the *Bacteroides* enterotype. Individuals with this enterotype are known to have low microbial gene richness (LGR), which is heavily associated with chronic systemic conditions and an increased risk of morbidities [24], whereas individuals with the *Prevotella* enterotype have a much higher gene richness (HGR). LGR boosts the proportion of pathobionts, resulting in increased intestinal permeability and inflammation [24]. It is also associated with increased mucus degradation, decreased butyrate-formation, higher oxidative stress, and reduced methane and hydrogen production, suggesting an inflammatory microbiota [25]. LGR also increases *Bacteroides* spp. that have genomic potential to produce detrimental metabolites, including modules that degrade aromatic amino acids and β-glucuronide [25].

## 6. Microbial Dysbiosis in Nutritional and Gastrointestinal Disorders and Cancers

While the high caloric diet characterizing the typical American lifestyle contributes to the onset of obesity, microbial dysbiosis has also been implicated in the development and persistence of the disease and its comorbidities, including diabetes. Due to the prevalence of the *Bacteroides* enterotype in Western culture, many American microbiotas possess LGR, which has been associated with obesity [25]. These individuals tend to gain significantly more weight and present with more evident inflammatory phenotypes in comparison to their HGR counterparts [25]. Conversely, HGR has been associated with marked decreases in adiposity measures, lower levels of circulating cholesterol, and decreased inflammation [26]. In addition, high-fat diets downregulate the tight junction proteins occludin and ZO-1, increasing intestinal permeability [27].

The link between high fat-induced GI dysbiosis and diabetes is strong, especially when considering that high-fat diets increase intestinal permeability, endotoxemia, and subsequently inflammation. While the current literature reveals only a moderate degree of species-specific GI dysbiosis in type 2 diabetes (T2D) patients, functional analysis shows alteration of metabolic pathways in T2D [28]. As such, the dysbiosis seen in T2D is better described as functional in nature rather than microbial. Among these altered functions are decreased butyrate synthesis, enrichment of sugar and branched-chain amino acid transporters, increased xenobiotic metabolism, and reduced bacterial chemotaxis and metabolism of vitamins and cofactors [28]. While the functional dysbiotic repercussions are more severe, there is an increase in opportunists linked to bacteremia and intra-abdominal infection such as *Escherichia coli*, *Clostridium hathewayi*, *Clostridium ramosum*, and *Clostridium symbiosum* [28]. *Clostridium* clusters XIVa and IV, known butyrate producers, were also negatively correlated with the rise of certain *Clostridium* spp. in T2D patients [28]. LGR individuals present with more pronounced insulin resistance, hyperinsulinemia, and elevated predisposition to diabetic conditions [25,26]. Table 2 summarizes the findings of several reports describing the importance of several GI microorganisms according to GI health status.

Chronic refractory inflammation of the alimentary canal with recurrency characterizes inflammatory bowel disease (IBD). The GI microbial profile of IBD patients reveals decreased microbial diversity, including fewer Firmicutes and *Bacteroides*, a relative increase in Enterobacteriaceae, and changes in microbial composition [23,28]. Furthermore, the concentrations of anti-inflammatory bacteria, including *Faecalibacterium prausnitzii*, a member of the butyrate-producing *Clostridium* cluster IV group [23,29,30], are typically decreased, whereas pro-inflammatory bacteria, such as adhesive/invasive *E. coli* (AIEC), are typically increased [23,29]. AIEC adheres to the intestinal epithelium, affecting its permeability and upregulating inflammation [31]. In addition, the number of mucolytic bacteria such as *Ruminococcus torques* and *Ruminococcus gnavus*, as well as sulfate-reducing bacteria such as *Desulfovibrio*, increase relatively and further promote inflammation and epithelial damage [23].

Increasing evidence has implicated microbes in the pathogenesis of certain cancers, especially CRC. Several mechanistic hypotheses have been proposed in regard to microbial participation in CRC. The “driver-passenger” theory states that certain bacterial drivers induce DNA damage in epithelial cells, thus initiating GI tumorigenesis and creating a tumor microenvironment that is more receptive to dysbiosis and subsequent colonization by further carcinogenic bacteria [18]. Another mechanism proposes that pro-carcinogenic bacteria precede an upregulation of inflammation, leading to oncogenesis [18]. Though the temporal relation between the two remains unknown, studies in murine models with altered inflammatory/immune responses indicate that dysbiosis can stimulate cancer development [32]. Several bacteria are suspected to aid in colorectal oncogenesis, specifically *Streptococcus bovis*, *Helicobacter pylori*, *Bacteroides fragilis*, *E. faecalis*, *Fusobacterium* spp., and *E. coli*. [18]. *S. bovis* was connected to enterococcal endocarditis of CRC origin [33], with linkage to GI disease and CRC [34]. *H. pylori*, a well-known cause of gastric cancer, has been associated with colorectal adenomas [35], and an increased chance of developing CRC [36], though its full role in CRC remains controversial. Higher levels of *B. fragilis* have been shown in patients with CRC [37], and its toxin can alter metabolic pathways, leading to increased cell proliferation, DNA damage, and pro-inflammatory cytokine release in murine models [18]. AIEC is implicated in CRC due to IBD’s role in the pathogenesis of CRC, and elevated levels have been observed in the colon of CRC patients and colorectal lesions when compared to regular colonic mucosa [38,39]. *F. nucleatum*, a bacterium strongly linked to periodontitis, has also been found in higher abundance in colorectal adenomas when compared to adjacent mucosa [40].

## 7. The Link between the Oral and Gut Microbiomes

Despite the fact that the oral and GI microbiomes contain varying types and amounts of bacterial species, their interconnected nature suggests potential routes of bacterial transfer. Two hypotheses have emerged for oral bacterial transmission to the gut: the hematogenous route, whereby oral bacteria enter lesions and systemically circulate to and colonize the GI mucosa, and the enteral route, where bacteria from the oral cavity travel through the stomach to the intestines. The human body possesses several defensive mechanisms and barriers against microbes, including neutralization via gastric acidity and colonization resistance against the enteral route; nevertheless, instances may exist whereby those barriers are lowered. Antibiotic use diminishes the concentration of the GI microbiota, and some oral bacteria such as *Klebsiella* spp., frequently found in IBD patients, are known to encode antibiotic-resistant genes [41], thus clearing the way for its colonization of the GI tract. In addition, patients with achlorhydria, commonly associated with long-term proton pump inhibition, contain elevated levels of oral bacteria in their GI system [42]. Some microbes, such as *P. gingivalis*, are even known to be acid-resistant, especially at higher inoculation doses [43]. Regardless of the route, evidence suggests that over half of bacterial species in the GI system undergo oral–gut translocation, even without pathology [44], though individuals who suffer from cirrhosis, CRC, and RA show more pronounced examples of oral to gut bacterial translocation [45,46,47]. Among the many known oral bacteria that can be found in the gut of patients with GI disease are members of the genera *Staphylococcus*, *Porphyromonas*, *Veillonella*, *Fusobacterium*, *Actinomyces*, and *Parvimonas* [42]. Table 2 summarizes the findings of several reports describing the importance of several gastrointestinal microorganisms according to GI health status.

**Table 2:** Importance of specific gastrointestinal (GI) microorganisms according to GI health status.

GI Health Status	GI Microbiota	Importance	Ref.
Health	Firmicutes, Bacteroidetes, Actinobacteria, Proteobacteria	A typical healthy GI microbiome contains 30–50% Firmicutes, 20–40% Bacteroidetes, 1–10% Actinobacteria, and a small percent of Proteobacteria.	[18]
	*Bacteroides* spp., *Parabacteroides* spp., *Prevotella* spp., *Desulfovibrio* spp., *Ruminococcus* spp., *Akkermansia* spp.	Three enterotypes have been discovered depending on diet: enterotype I (*Bacteroides* spp., *Parabacteroides* spp.) with carbohydrate, fat, and protein-based diets, enterotype II (*Prevotella* spp., *Desulfovibrio* spp.) with high-fiber diets, and enterotype III (*Ruminococcus* spp., *Akkermansia* spp.) which is the most commonly observed.	[24]
Dysbiosis	Enterobacteriaceae	These bacteria are commonly associated with oxidative stress within the gut and relative increases in the proportion of these microbes can be found in individuals with inflammatory bowel disease, especially colitis	[23]
	*Bacteroides* spp.	In individuals with low-gene richness, *Bacteroides* spp. increase non-proportionally and have the genomic potential to secrete metabolites that negatively impact the host.	[25]
Obesity	*Bacteroides* spp.	The dominance of *Bacteroides* spp. in enterotype 1 leads to low-gene richness within the microbiota, which in-turn correlates with obesity, increased inflammation, and significantly higher levels of weight gain.	[25]
Diabetes	*Escherichia coli, Clostridium hathewayi*, *Clostridium ramosum*, *Clostridium symbiosum*	These are examples of opportunistic pathogens which are linked to bacteremia and intra-abdominal infections as a result of diabetic dysbiosis.	[28]
Inflammatory Bowel Diseases	*Escherichia coli (AIEC), Ruminococcus torques, Ruminococcus gnavus,* Enterobacteriaceae, *Desulfovibrio* spp.	In patients with IBD, these species have been shown to promote inflammation and increase mucus degradation, damaging epithelial cells and increasing intestinal permeability. AIEC has been shown to be present in 38% of individuals with active Crohn’s disease compared to 6% in healthy control subjects.	[23]
GI Cancer	*Streptococcus bovis*, *Helicobacter pylori*, *Bacteroides fragilis, Enterococcus Faecalis, Fusobacterium* spp., *Escherichia coli (AIEC)*	These species are suspected to aid in colorectal oncogenesis with relative risks of colorectal cancer and prevalence of other diseases (endocarditis, gastric cancer, periodontitis) increasing as well.	[18]
	*Fusobacterium nucleatum*	*Fusobacterium nucleatum* has demonstrated an ability to colonize the GI tract and further promote microbial dysbiosis and subsequently colorectal cancer. Identical clones of oral *Fusobacterium nucleatum* have been isolated from the colorectal cancer lesions of patients with this disease.	[47]

## 8. Newly Emerging Connections between Immune-Mediated Inflammatory Disease and Microbial Dysbiosis

Systemic lupus erythematous (SLE) is a chronic autoimmune disease involving multiple systems in the body and presents with a heterogeneity of symptoms. The pathogenesis of SLE is driven mainly by antibodies and immune complexes directed toward nuclear peptides, dsDNA of the nucleosome, and Sjogren’s syndrome (SS)-related antigen A (SSA, Ro), which are produced by autoreactive B-cells. Recent and newly emerging evidence has elucidated the involvement of both oral and GI microbial dysbiosis in SLE and SS [48,49,50,51,52,53].

Lower Firmicutes:Bacteroidetes ratios were found in GI microbiomes of individuals with SLE compared to healthy controls, leading to increased glycan production and oxidative phosphorylation [52,53]. Similarly, the Firmicutes:Bacteroidetes ratio is also decreased in patients with SS, indicating a possible role in host immune modulation and autoimmunity [53]. Bacteroidetes are responsible for the production of SCFAs such as butyrate, which is associated with a healthy GI tract, and the alteration of SCFA production is associated with intestinal dysbiosis in SLE [54]. The reduced availability of SCFAs can induce a state of inflammation through clonal expansion of TH-17 cells, leading to recruitment of additional proinflammatory cytokines (e.g., IL-6, IL-7, IL-21, IL-23) [48]. This culminates in a breakdown of the mucosal barrier and exposure of the host immune system to new antigens that, through molecular mimicry, could lead to cross-reactivity towards host antigens and stimulate an autoimmune response [48].

In patients with SLE, a decreased diversity of oral microbial species is illustrated by increased numbers of *Selenomonas*, *T. denticola*, *Veillonella*, and *Leptotrichia* that directly correlate with elevated levels of inflammatory cytokines IL-6, IL-17, and IL-33 [49]. In patients with primary SS, salivary *Bifidobacterium*, *Dialister*, and *Lactobacillus* levels were elevated, while *Leptotrichia* abundance was reduced [55]. Studies have also found lower Proteobacteria numbers and alpha diversity in the salivary microbiome of patients with SS and increased levels of *Veillonella* and *Fusobacterium* [51]. *Veillonella parvula* could be a potential biomarker for the early detection of SS [56]. *P. gingivalis* can induce reactivation of Epstein–Barr virus (EBV), a virus that is strongly implicated in the pathogenesis of both SLE and SS [48,57]. EBV antigens from the viral lytic phase resemble SLE antigens and could stimulate auto-reactivity through molecular mimicry [58]. EBV has also been found in salivary glands of patients with SS and could potentially contribute to the activation and differentiation of B cells toward autoreactivity [58].

Another autoimmune condition linked to microbial dysbiosis is RA, a chronic inflammatory condition affecting the synovial membrane of joints. The autoantibodies to rheumatoid factor as well as anticitrullinated protein antibodies (ACPA) are characteristic immune responses in this disease [50]. Dysbiosis occurs in both oral and gut microbiomes in patients with RA [50]. Periodontitis, a known risk factor for RA, may be involved in the development of ACPA [48,50].

One periodontal pathogen implicated in the pathogenesis of RA is *P. gingivalis*. This bacterium possesses two enzymes: peptidyl arginine deaminase, which can convert arginine into citrulline in bacterial and human proteins, and arginine gingipain, which creates a C-terminal arginine and enables *P. gingivalis* to citrullinate human fibrinogen and alpha-enolase [50]. Although citrullinated proteins are common, antibodies against human citrullinated alpha-enolase show cross reactivity with *P. gingivalis* enolase and could be a potential source for autoimmunity directed against ACPAs [48,50]. Additionally, patients with RA have elevated antibodies to periodontal pathogens (*P. gingivalis*, *P. intermedia*, *T. forsythia*), which correspond to increased serum levels of ACPA and C-reactive protein [59]. DNA from periodontal pathogens, such as *P. gingivalis* and *T. forsythia*, have also been found in synovial fluid isolates, and these pathogens can induce cytokine (IL-1, IL-6, TNF-a) production by monocytes via TLR-9 receptor [50]. Table 3 summarizes the findings of several reports describing the links between and significance of oral and gastrointestinal microorganisms and specific systemic diseases.

Several shifts in microbial biomes of RA patients have been elucidated through sequencing and some shifts in organism content are conserved between oral and GI sites. Notably, levels of *Haemophilus* species were decreased in both oral and GI microbiomes while *Lactobacillus salivarius* was elevated in these biomes. Furthermore, there was a covariation between particular genera subsets in oral and gut microbiomes that was conserved across individuals with RA, potentially revealing a complex interaction between the oral and GI microbiomes [46]. Patients with new onset RA have a characteristic elevation in numbers of *Prevotella* species in both GI and oral microbiomes, a marked decrease in *Bacteroides* in GI microbiomes, and elevated levels of *Leptotrichia* genus in oral microbiomes [46,60]. Specifically, the presence of *Prevotella copri* in new onset RA individuals could implicate this microorganism in the pathogenesis of RA or serve as a potential marker of disease [60].

## 9. Microbial Dysbiosis in the Oral Cavity Leading to Dysbiosis in the Gastrointestinal System

**Figure 1:**
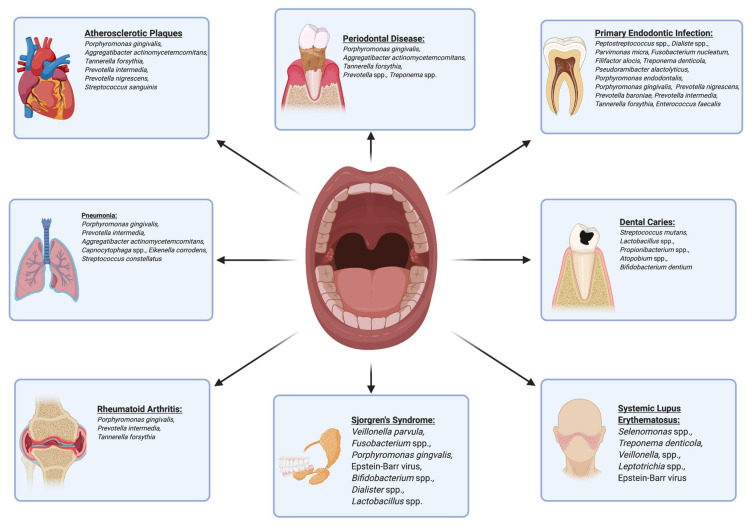
Oral bacteria are linked to numerous oral and systemic diseases, highlighting the importance of oral microbial homeostasis in the maintenance of health and prevention of pathology.

With increasing evidence suggesting oral bacterial translocation to the gut, it becomes imperative to assess whether microbial dysbiosis in the oral cavity can precipitate dysbiotic conditions in the GI tract that can trigger systemic disease. Periodontitis, the most prominent disease caused by oral dysbiosis, is characterized by several key bacterial species such as *F. nucleatum*, *A. actinomycetemcomitans*, and *P. gingivalis*. Upon analysis, the oral microbiota of CRC patients was distinct and predictive, showing prominent oral *F. nucleatum* levels [61] with subsequent studies confirming the presence of identical clones of oral *F. nucleatum* in CRC lesion biopsies [47]. Oral administration of *A. actinomycetemcomitans* in conjunction with high-fat diets in mice showed signs of non-alcoholic fatty liver disease (NAFLD) exacerbation through dysbiotic GI changes as well as higher insulin resistance and glucose intolerance [62]. Though the significance of these microorganisms to GI dysbiosis is apparent, *P. gingivalis* may represent one of the clearest connections between oral and GI dysbiosis. Oral administration of *P. gingivalis* significantly increased endotoxemia and reduced mRNA expression of ZO-1, occludin, and Tjp1 tight junction proteins in the small intestine [63,64]. Plasma analysis revealed elevated levels of bacterial DNA, except for *P. gingivalis*, which suggests that the observed endotoxemia was not due to *P. gingivalis* in the bloodstream, but rather its effect on the microbiota of the GI system [63]. Furthermore, even a single administration of oral *P. gingivalis* can increase the prevalence of Bacteroidetes while decreasing the abundance of Firmicutes [64]. Just as *P. gingivalis* acts as an oral keystone species, its effect in the GI system is magnified despite its low abundance. Upon single oral administration of *P. gingivalis*, significant changes in tight junction protein expression and the GI microbiota were seen, despite the fact that less than 0.003% of the bacterial load in fecal samples belongs to the Porphyromonadaceae family [64]. The presence of *P. gingivalis* in the GI microbiota has been linked with a milieu of inflammatory/autoimmune diseases associated with GI dysbiosis including RA and NAFLD [65], warranting further research into the connection between microbial dysbiosis found between the oral and GI systems and potential therapeutic targets that expose this connection for preventative medicine. Figure 1 highlights the importance of oral microbial homeostasis in the maintenance of health and in the prevention of pathology.

## 10. Therapeutics for Reversing Microbial Dysbiosis—Fecal Microbiota Transplantation

The goal for future therapeutics in reversing microbial dysbiosis is to establish personalized and targeted treatments, with the primary objectives being the eradication or reduction in disease-associated microbiota and rehabilitation of health-associated microbiota. Recent research concerning methods of correcting microbial dysbiosis build upon the existing knowledge of fecal microbiota transplantation (FMT), probiotics, prebiotics, and synbiotics while simultaneously exploring newer innovative therapies including precision medicine.

FMT is a therapy performed by acquiring a stool sample from a healthy individual and transferring the sample to a patient experiencing microbial dysbiosis of the gut. The goal in utilizing a microbial sample from a healthy individual is to introduce microbes associated with health and promote a shift in the microbiota of the patient experiencing microbial dysbiosis. FMT has a history of success in the treatment of recurrent *C. difficile* infections [66]. Numerous studies provide evidence that FMT treatment for *C. difficile* infections results in disease reduction, with a success rate of over 90% [66]. The success of FMT in treating the microbial dysbiosis state of *C. difficile* infections suggests the potential use of FMT in other dysbiosis-associated disease states.

Studies utilizing FMT for functional improvement in patients with metabolic conditions show promising initial findings, including the treatment of obesity and diabetes. FMT from lean donors to individuals with metabolic syndromes demonstrated an improvement in insulin sensitivity, increased levels of SCFA-producing bacteria, including *Roseburia intestinalis* and *Eubacterium hallii*, and produced general increases in gut microbial diversity six weeks after infusion [67,68]. Relative increases in health-associated *Bifidobacterium*, *Lactobacillus salivarius*, *Butyrivibrio*, and *Eubacterium* were observed following treatment [68]. Insulin sensitivity returned to the pre-treatment level at 18 weeks, suggesting that multiple rounds of FMT are necessary for long-term stable solutions [68].

FMT has also been used therapeutically as a treatment for patients with IBD, including both ulcerative colitis (UC) and Crohn’s disease (CD). One meta-analysis found a total remission rate of 45% for IBD following FMT, with a 22% clinical remission rate for patients with UC and 60.5% remission rate for patients with CD [69]. These results suggest that FMT has a clinical impact on IBD, necessitating further analysis of exact microbial shifts.

A summary of three cases describing patients with multiple sclerosis (MS) and accompanying severe chronic constipation found that these individuals experienced significantly improved GI and neurological symptoms following FMT [70]. Based on these results, it was speculated that a specific GI pathogen may have contributed to MS neurologic symptoms. A case report examining a patient with idiopathic thrombocytopenic purpura and UC found that FMT resulted in a reduction in UC symptoms and a return to a normal platelet count [71]. These case reports suggest a role of gut microbes in eliciting the symptoms of inflammatory systemic disorders and use of FMT in reversing dysbiosis. Table 4 describes the findings of several reports describing FMT as a therapeutic strategy for the reversal of microbial dysbiosis.

## 11. Therapeutics for Reversing Microbial Dysbiosis—Probiotics, Prebiotics, and Synbiotics

Probiotics, prebiotics, and synbiotics have been extensively used in the treatment of various GI and systemic diseases. Probiotics administer specific live bacterial species through oral ingestion to introduce health-associated bacteria to the GI microbiota. Prebiotics are composed of non-digestible carbohydrates that serve as selective dietary substrates utilized by health-associated bacteria and can cause specific changes in the composition of the gut microbiota. Synbiotics are a combination of probiotics and prebiotics, including both live bacterial species and the dietary substrates needed for their growth.

Probiotics have been shown to impact systemic metabolic disorders including diabetes, obesity, hypertension, and hyperlipidemia. Probiotic supplementation with *Lactobacillus* and *Bifidobacterium* to patients with T2D resulted in lower HbA1C scores and decreased LDL and total cholesterol levels [72]. In patients with a body mass index greater than 25, probiotic administration of multiple *Streptococcus*, *Lactobacillus*, and *Bifidobacterium* species resulted in weight reduction and a significant increase in *Lactobacillus plantarum* in the gut microbiota [73]. A meta-analysis of pre-hypertensive and hypertensive patients given probiotic-fermented milk with various strains of *Lactobacillus* was associated with significantly reduced systolic and diastolic blood pressures [74]. A study of probiotic supplementation with several strains of *Lactobacillus*, *Bifidobacterium*, and *Streptococcus* found significant shifts in the gut microbiota following probiotic treatment, namely an overall increase in the total aerobes and anaerobes, *Bifidobacterium*, *Lactobacillus*, and *Streptococcus* levels. Conversely, a reduction in *Bacteroides*, Coliforms, and *E. coli* was also observed [75].

Supplementation with various prebiotic and synbiotic combinations has also been found to improve physiologic and biochemical markers of diabetes, hyperlipidemia, obesity, and hypertension. Supplements used include oligofructose, fructo-oligosaccharides, galacto-oligosaccharides, inulin, *Lactobacillus*, and *Bifidobacterium* [76]. Information on exact microbial shifts following treatment is lacking, necessitating further investigation in this area.

Probiotics and prebiotics are also utilized for the treatment of IBD, including CD and UC. Prebiotic studies utilizing fructo-oligosaccharides and galacto-oligosaccharides in patients with CD found a significant increase in *Bifidobacterium* populations, a species associated with health [77]. A synbiotic combination of microbes and substrates, including *Lactobacillus*, *Bifidobacterium*, *Streptococcus*, inulin, and oligofructose in patients with irritable bowel syndrome (IBS) resulted in improved IBS symptoms and an increased presence of *Lactobacillus acidophilus* and *Bifidobacterium animalis* in feces one week following treatment [78].

Multiple autoimmune and inflammatory disorders have been linked to gut microbial dysbiosis, including SLE, MS, and RA, for which the therapeutic use of probiotics and prebiotics could potentially be beneficial. Probiotic treatment of RA with multiple species of *Lactobacillus* and treatment of SLE with *Bifidobacterium* and *Lactobacillus* were both found to be efficacious in reducing inflammatory mediators and symptoms in their respective disease states [79,80]. MS treatment with probiotic supplementation of *Lactobacillus* resulted in a microbial shift, decreasing populations of MS-associated *Anaeroplasma*, Rikenellaceae, and *Clostridium* [81]. Probiotic supplementation with multiple *Lactobacillus*, *Streptococcus*, and *Bifidobacterium* species resulted in an increased abundance of *Lactobacillus* and *Bifidobacterium* in the gut and produced an anti-inflammatory peripheral immune response in patients with MS [82].

**Figure 2:**
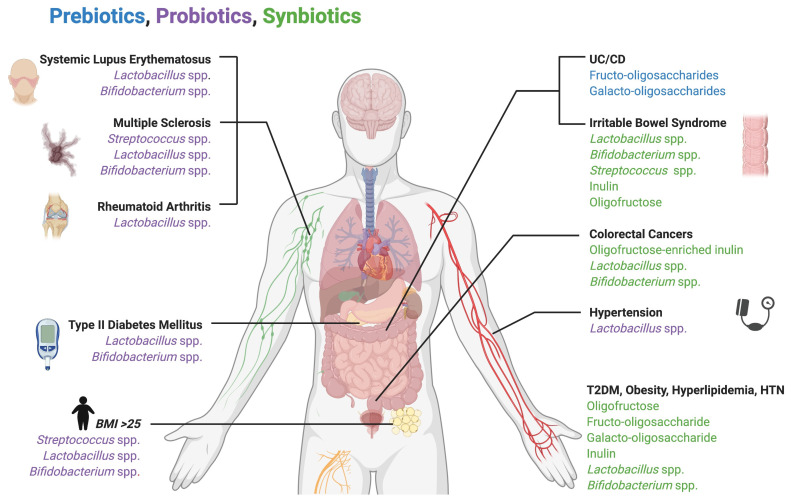
Use of prebiotics, probiotics, and synbiotics as therapeutic strategies for the reversal of microbial dysbiosis. Numerous bacterial species and dietary substrates have been shown to therapeutically shift microbiota composition and alleviate the symptoms and biomarkers of systemic diseases.

Potential risk reduction in CRC following synbiotic supplementation was suggested in a study of oligofructose-enriched inulin, *Lactobacillus*, and *Bifidobacterium* in which several CRC biomarkers, including CEA and CA19-9 tumor markers, improved and significant changes in fecal flora were produced, including increased *Bifidobacterium* and *Lactobacillus* populations and decreased *Clostridium perfringens* [83,84]. Additionally, treatment of CRC with supplementation of inulin-type fructans was shown to inhibit growth and induce apoptosis in colon tumor cells [85]. Figure 2 summarizes the use of prebiotics, probiotics and synbiotics as therapeutic strategies for the reversal of microbial dysbiosis.

## 12. Targeted Therapy for Dysbiosis in Systemic Diseases

Analysis of the gut and oral microbiome through metagenomic shotgun sequencing has revealed a specific microbial dysbiotic profile associated with RA [46]. This is in accordance with the results of previous 16s rRNA sequencing studies analyzing fecal and oral samples of RA patients. The results indicate that there is a significant reduction in *Porphyromonas*, *Bacteroides*, *Prevotella*, *Clostridium*, and *Bifidobacterium* groups, and an upsurge of *Lactobacillus* communities in these patients as compared with healthy individuals. These patients were then treated with disease-modifying antirheumatic drugs and a marked reduction in RA-associated microbes was observed in dental plaques, especially in patients who saw a greater reduction in RA symptoms after treatment. Additionally, patients treated with a combination of methotrexate and T2 glycosides isolated from *Tripterygium wilfordii* showed a reduction in RA-associated gut microbiota.

The concept of precision editing of microbiotas involves targeting specific dysbiotic organisms to inhibit their pathogenicity while having little effect on the complete microbiome. This model has been used in rodents to ameliorate inflammatory diseases such as infectious colitis [86]. It is well-documented that overgrowth of Enterobacteriaceae is common in colitis, and this may contribute significantly to the disease state [87]. A strong association has been found with a molybdopterin cofactor (MoCo) pathway and rapid proliferation of colitis-associated Enterobacteriaceae, suggesting that this pathway greatly enhances the fitness of these organisms in an inflammatory environment. Furthermore, it has been shown that tungsten-enriched media inhibit this MoCo pathway by competing for the active site of the molybdopterin cofactor [88]. This theory was tested on colitis-affected mice by administration of tungstate [86], which caused a significant microbial shift from a dysbiotic profile to a more normalized state. Moreover, there was a significant reduction in inflammatory markers present in mice treated with this therapy without a marked effect on microbial composition in non-inflammatory states. Of note, both the host and other microbial taxa were spared any negative effects.

Maternal high-fat diets in conjunction with obesity have also been associated with behavior and mental health disorders such as major depressive disorder, generalized anxiety disorder, attention deficit hyperactivity disorder and autism spectrum disorder (ASD) [89]. Remarkably, a characteristic microbial dysbiosis of the gut is seen in both the mother and offspring of individuals with the aforementioned diseases when obesity is involved [90]. In one experiment, two groups of female mice were fed either a high-fat diet or regular diet and were then bred [91]. When compared to the offspring of mice on a regular diet, the offspring of the high-fat diet mice exhibited social deficits, displaying antisocial behavior and reduced interaction with others. Fecal samples of these mice showed a marked reduction in microbial diversity in the gut and a nine-fold reduction in *Lactobacillus reuteri*, an important producer of oxytocin, compared to the control. Testing of oxytocin-immunoreactive neurons revealed reduced levels in offspring of mice fed a high-fat diet. Reintroduction of *L. reuteri* to these progenies however eliminated social deficits and restored levels of oxytocin-immunoreactive neurons. Interestingly, co-housing of the control offspring with descendants of the mice fed high-fat diets ameliorated social deficits and restored their microbiomes to one more closely resembling the control. Table 5 describes the findings of several reports which examine the targeted therapy for dysbiosis in systemic diseases.

## 13. Targeted Therapy for Dysbiosis in Oral Diseases

Symbiotic diversity of oral flora is essential for oral health; thus, the use of broad-spectrum antibiotics which can alter the entire microbial flora must shift to that of a more personalized and targeted approach. In this manner, specific therapeutic methods can be used to target the etiology of dysbiosis, which would require an understanding and classification of individuals based on biomarkers and microbial ecology. One method proposes classifying individuals into categorical oral ecotypes [92]. When saliva samples were taken from subjects and analyzed, it was found that a predisposition for caries development was characterized by increased levels of lipid degradation products, decreased salivary pH, and low salivary microbial diversity with a prevalence of saccharolytic microbes. Conversely, individuals with increased salivary pH, decreased lysozyme activity, and a prevalence of proteolytic microorganisms were predisposed toward periodontal disease and gingival inflammation [93].

*S. mutans* is widely considered to be a keystone pathogen in the development of caries. Two key virulence factors of this organism are the PAc surface adhesin protein (Antigen I/II, P1), and glucosyltransferases (GTFs) used to generate glucans from sucrose [94]. Efforts to develop precision therapy against these virulence factors in oral *S. mutans* have shown promising results. Immune complex administration through an anti-Antigen I/II monoclonal antibody named “Guy’s 13 plantibody” elicited the formation of anti-adherence antibodies in mice and promoted a statistically significant inhibition of *S. mutans* adherence [95]. The caries rate was also reduced upon administration of polyclonal IgG antibodies to GTFs and glucan-binding proteins (GBPs) [96].

Individuals with an inclination toward a low pH and cariogenic ecotype may benefit from the introduction of *Streptococcus dentisani* [97]. This novel strain from the *S. mitis* group was cultured from the dentition of caries-free individuals and has been found to raise the pH in the oral environment through the breakdown of arginine and subsequent production of ammonia [98]. Furthermore, supernatants derived from *S. dentisani* were found to inhibit growth of many pathogenic oral microorganisms, including *S. mutans*, *Streptococcus sobrinus*, *F. nucleatum*, and *P. intermedia* [99]. Moreover, scanning electron microscopy imaging of the supernatant-treated cells showed cell wall structural changes in *P. intermedia*, pore formation in *S. mutans*, and even cell lysis of *F. nucleatum*.

Broad-spectrum antimicrobial mouthwashes such as chlorhexidine are often used to control dysbiosis. However, a novel decapeptide called KSL (KKVVFKVKFK–NH2) demonstrated desirable impacts in oral environments [99]. When plated with *S. mutans*, KSL showed significant antimicrobial effects and inhibited biofilm formation as well. The peptide also had antifungal properties against *Candida albicans*.

With the established relationship between systemic disease and bacteria involved in periodontitis such as *P. gingivalis* and *F. nucleatum*, novel precision therapeutic approaches are being explored to assist host immune response against these pathogens. Antimicrobial peptides may prove to be an effective approach in restoring oral health. Sheep myeloid antimicrobial peptides are cathelicidins isolated from sheep bone marrow that demonstrate antimicrobial effects [100]. A specific 29-amino acid peptide called SMAP29 and a more potent SMAP28 exhibited antimicrobial activity against *P. gingivalis* and *F. nucleatum*; however, the activity was broad-spectrum and also active against other bacteria such as *A. actinomycetemcomitans*, *S. mutans*, *S. sanguinis*, *Actinomyces israelii*, and *Actinomyces naeslundii*. Efforts to increase the specificity of this peptide included conjugation with immunoglobulin G (IgG) antibodies directed toward the cell surface of *P. gingivalis* [101]. The effects on specificity were found to be concentration-dependent, with the 20 μg protein/mL concentration being more specific and sparing *A. actinomycetemcomitans* and *Peptostreptococcus micros,* as compared to the 50μg protein/mL concentration, which quickly and non-selectively killed *P. micros*, *A. actinomycetemcomitans*, and *P. gingivalis* [101]. Application of this precision therapy against keystone periodontitis pathogens may help to eliminate disease with the retention of commensal diversity. Table 6 describes the implications of several targeted therapies aimed at rectifying oral microbial dysbiosis.

## 14. Concluding Remarks, Limitations and Future Directions

The balance between eubiotic health and dysbiotic pathology is dependent upon the diversity and quantity of specific microorganisms in the host microbiome. The interconnection between oral and systemic dysbiosis provides a common pathway for progression to autoimmune, inflammatory, and pernicious diseases. Conventional treatment modalities for these diseases often come with adverse effects. The development of novel targeted therapeutics such as FMT, biotic modulation, and precision medicine has demonstrated favorable results in reestablishing a healthy microbiota or immune state. Additional research will need to be conducted in the disciplines of genomics, pharmacology, and microbiology to enhance our understanding of disease pathogenesis and resolution. Although half of the bacterial species found in the GI tract have oral origins, further research that targets quantitative assessment of the oral/GI translocation may further elucidate the characteristics, etiopathogenesis, and link between oral inflammatory pathologies and systemic diseases [102]. One critical limitation in the field of microbiome therapeutics is the existence of host variability in humans, due to differences in human lifestyle and genetics that can influence microbiota composition; this inter-person heterogeneity may potentially lead to incorrect associations to disease and would require the acquisition of exact match control groups to make accurate comparative associations [103]. Still in its infancy, precision medicine has great potential to be further adopted in clinical practice, leading to improved treatment efficacy and greater efficiency in therapeutics. Antibiotics are currently used for the treatment of many bacterial-associated illnesses, but are broad-spectrum and reduce both pathologic and health-associated bacteria. Several methods are being explored that can achieve highly specific targeting and elimination of bacteria, including the use of bacteriocins, bacteriophages, and engineered phage therapy [104]. Subtractive genomics has also been used to identify protein targets essential for the survival of specific microorganisms, including *F. nucleatum* and *C. albicans,* and may be exploited in future research in the design of targeted inhibitor drugs [105,106]. Next-generation probiotics are now being developed and commercialized as live biotherapeutics for exclusive use in pharmaceutical, non-food applications [107]. Further challenges in the development of microbiome therapeutics could include the design of therapies affecting the microbiome of specific anatomical regions, perhaps only the oral microbiome, as well as the development of permanent or stable eubiotic microbiomes for consistency in health, and the identification of microbial biosensors for disease [104]. Multidisciplinary collaboration will hopefully reveal further potential for precision medicine as a targeted therapy, which could shift the management of long-term complications of systemic disease to a more personalized treatment modality.

## Figures and Tables

**Table 1:** Importance of specific oral microorganisms according to oral health status.

Oral Health Status	Oral Microbiota	Importance	Reference
Health	*Streptococcus mitis*, *Streptococcus salivarius*, *Streptococcus sanguinis*	Initially, various *Streptococcus* spp. are acquired as pioneer species and begin to modulate pH and nutrient availability in the oral cavity, setting the tone for subsequent colonization by other members of the oral microbiota.	[4]
	*Fusobacterium* spp., *Granulicatella* spp., *Neisseria* spp., *Haemophilus* spp., *Corynebacterium* spp., *Rothia* spp., *Actinomyces* spp., *Prevotella* spp., *Capnocytophaga* spp., *Porphyromonas* spp.	These phylotypes are commonly associated with healthy oral microbiomes and monitoring relative numbers of these species can indicate a change in homeostatic balance.	[6]
Caries	*Streptococcus mutans*	*Streptococcus mutans* can resist high levels of oxidative stress and in-turn has the ability to out-compete other microorganisms under conditions of high carbohydrate metabolism. These abilities change the homeostatic balance of the oral microbiome and allow *Streptococcus mutans* to be the primary driver of carious lesions.	[7]
	*Lactobacillus* spp., *Propionibacterium* spp., *Atopobium* spp. *Bifidobacterium dentium*	The listed microbes thrive in low-pH conditions even when *Streptococcus mutans* is not present. Carious lesions lacking *Streptococcus* *mutans* reported higher levels of *Bifidobacterium dentium* and *Lactobacilllus* spp.	[8]
Periodontitis	*Aggregatibacter actinomycetemcomitans*, *Treponema* spp., *Prevotella* spp. *Tannerella forsythia, Treponema denticola, Porphyromonas gingivalis*	Bacteria listed to the immediate left are all associated with periodontitis. *Porphyromonas gingivalis, Tannerella forsythia,* and *Treponema denticola* are all part of the “red complex,” which when present in large numbers, implicate a dysbiotic shift to periodontitis.	[4] [11]
Endodontic Infection	*Peptostreptococcus* spp., *Dialister* spp., *Parvimonas micra*, *Fusobacterium nucleatum, Filifactor alocis, Treponema denticola*, *Pseudoramibacter alactolyticus*, *Porphyromonas endodontalis*, *Porphyromonas gingivalis*, *Prevotella nigrescens, Prevotella baroniae, Prevotella intermedia*, *Tannerella forsythia, Enterococcus faecalis*	Primary infection of the pulp chamber commonly features the following species, while secondary infection of root canals is typically due to elevated levels of *Enterococcus faecalis*, *Filifactor alocis, Pseudoramibacter alactolyticus, Parvimonas micra*, *Propionibacterium propionicum*, *Streptococcus constellatus, and* *Streptococcus anginosus.*	[12]

**Table 3:** Link between and significance of oral and GI microorganisms and specific systemic diseases.

Disease	Link to Oral/GI Microbiota	Significance	Ref.
Atherosclerotic Plaques	*Porphyromonas gingivalis, Aggregatibacter actinomycetemcomitans, Tannerella forsythia, Prevotella intermedia, Prevotella nigrescens*, *Streptococcus sanguinis*	These bacteria have been found in atherosclerotic plaque samples. *Porphyromonas gingivalis* and *Aggregatibacter actinomycetemcomitans* have shown high levels of inflammatory immune response and presence of these bacteria may lead to a significantly increased risk for developing coronary artery disease.	[14]
Pneumonia	*Porphyromonas gingivalis, Prevotella intermedia, Aggregatibacter actinomycetemcomitans, Capnocytophaga* spp., *Eikenella corrodens, Streptococcus constellatus*	These bacteria are thought to play direct roles in the pathogenesis of pneumonia.	[15]
Systemic Lupus Erythematosus (SLE)	*Selenomonas* spp., *Treponema denticola, Veillonella* spp., *Leptotrichia* spp.	Salivary levels of the following microorganisms have been shown to increase in patients with SLE and correlate directly with increased levels of inflammatory cytokines.	[49]
Systemic Lupus Erythematosus (SLE)/ Sjogren’s Syndrome (SS)	Lower Firmicutes to Bacteroidetes ratio	A lower Firmicutes to Bacteroidetes ratio has been shown in patients with SLE/SS and potentially increases inflammation.	[53]
	Epstein–Barr virus (EBV)	EBV lytic phase antigens may be responsible for activation of SLE/SS immune responses creating auto-reactive antibodies.	[48]
Sjogren’s Syndrome (SS)	*Bifidobacterium* spp., *Dialister* spp., *Lactobacillus* spp., *Leptotrichia* spp.	The first three bacteria are increased in salivary concentration for cases of primary SS. *Leptotrichia* spp. abundance was reduced in primary SS.	[55]
	*Veillonella parvula, Fusobacterium* spp.	These bacteria have also shown elevated concentrations in patients with SS, with *Veillonella parvula* showing promise as a biomarker in the early detection of SS.	[56]
Rheumatoid Arthritis (RA)	*Porphyromonas gingivalis*	Antibodies against human citrullinated alpha-enolase show cross reactivity with *Porphyromonas gingivalis* enolase and could be a potential source for autoimmunity directed against anticitrullinated protein antibodies (ACPAs).	[50]
	*Porphyromonas gingivalis*, *Prevotella intermedia*, *Tannerella forsythia*	Patients with RA have elevated antibody levels against periodontal pathogens which correspond to increased serum levels of ACPAs and C-reactive protein.	[59]

**Table 4:** Fecal microbiota transplantation (FMT) as a therapeutic strategy for reversal of microbial dysbiosis.

Condition Treated by Fecal Microbiota Transplantation (FMT)	Findings	References
Recurrent *Clostridium difficile* infections	Studies provide evidence that FMT treatment for *Clostridium difficile* infections results in disease reduction with a success rate of approximately 92%.	[66]
Metabolic conditions including obesity and diabetes mellitus	Improved insulin sensitivity. Increased levels of short-chain fatty acid producing bacteria. Increased *Roseburia intestinalis* and *Eubacterium hallii* (butyrate-producing bacteria). Increased gut microbial diversity. Increased health-associated *Bifidobacterium* spp., *Lactobacillus salivarius, Butyrivibrio* spp., and *Eubacterium* spp.	[67,68]
Inflammatory Bowel Diseases (IBD) including Ulcerative Colitis (UC) and Crohn’s Disease (CD)	45% entered disease remission; 22% clinical remission rate for patients with UC and 60.5% remission rate for patients with CD.	[69]
Autoimmune diseases: Multiple Sclerosis (MS) Idiopathic thrombocytopenic purpura (ITP) Ulcerative Colitis (UC)	Significantly improved gastrointestinal and neurological symptoms (MS) following FMT. Reduction in UC symptoms and an increase in platelet count to a normal level in patients with ITP.	[70,71]

**Table 5:** Targeted therapy for dysbiosis in systemic diseases.

Disease	Targeted Therapy for Dysbiosis in Systemic Diseases	Implication	Ref.
Rheumatoid arthritis (RA)	Disease modifying antirheumatic drugs Combination of methotrexate and T2 glycosides	Genetic and microbial sequencing in RA patients may provide foresight into treatment efficacy.	[46]
Colitis	Precision editing of microbiota Administration of tungstate	Specific targeting of dysbiosis causing microorganisms by inhibiting their pathogenicity and virulence factors Little effect on the complete microbiome. Host and other microbial taxa spared of negative effects.	[86]
Autism Spectrum Disorder (ASD)	Introduction of *Lactobacillus reuteri*	Precision medicine in ASD may lead to development of new strategies to rebalance the microbiome of these individuals, and perhaps reduce the associated morbidities.	[91]

**Table 6:** Targeted therapy for dysbiosis in oral diseases.

Disease	Targeted Therapy for Dysbiosis in Oral Diseases	Implication	Reference
Caries	Immune complex administration via Guy’s 13 plantibody	Targeted therapy of *Streptococcus mutans* via inhibition of glucosyltransferases and PAc surface adhesin protein.	[94]
	Introduction of *Streptococcus dentisani*	Novel strain from the *Streptococcus mitis* group shown to raise oral pH via breakdown of arginine into ammonia.	[98]
	Introduction of *Streptococcus dentisani*	Supernatants derived from *Streptococcus* *dentisani* have shown ability to inhibit pathogenic oral microorganisms including: *Streptococcus* *mutans, Streptococcus sobrinus, Fusobacterium nucleatum,* and *Prevotella intermedia.*	[97]
	Introduction of KSL decapeptide (KKVVFKVKFK–NH2)	KSL shows significant antimicrobial effects inhibiting biofilm formation of *Streptococcus* *mutans* and displaying antifungal properties against *Candida albicans*.	[99]
Periodontitis	Introduction of SMAP 29 and SMAP 28 (Sheep myeloid antimicrobial peptides)	SMAP29 and the more potent SMAP28 exhibit non-specific antimicrobial properties targeting *Porphyromonas gingivalis* and *Fusobacterium nucleatum* among many others.	[100]
	Introduction of IgG in combination with SMAPs	To increase specificity towards *Porphyromonas gingivalis*, specific IgG antibodies can be conjugated with SMAPs at a therapeutic concentration of 20 μg protein/mL.	[101]

## Data Availability

Data sharing is not applicable to this article.
